# Mortality in individuals with childhood ADHD or subthreshold symptoms – a prospective perinatal risk cohort study over 40 years

**DOI:** 10.1186/s12888-022-03967-3

**Published:** 2022-05-09

**Authors:** Nella Schiavone, Maarit Virta, Sami Leppämäki, Jyrki Launes, Ritva Vanninen, Annamari Tuulio-Henriksson, Ilkka Järvinen, Eliisa Lehto, Katarina Michelsson, Laura Hokkanen

**Affiliations:** 1 Department of Psychology and Logopedics, University of Helsinki, Helsinki, Finland; 2 Department of Psychiatry, Helsinki University Hospital, Helsinki, Finland; 3 Department of Clinical Radiology, Kuopio University Hospital and School of Medicine, Clinical Radiology, University of Eastern Finland, Kuopio, Finland; 4 Children’s Hospital, Helsinki University Hospital, Retired, Helsinki, Finland

**Keywords:** ADHD, Subthreshold ADHD, Adult, Mortality, Cohort, Perinatal risk

## Abstract

**Background:**

Attention-deficit/hyperactivity disorder (ADHD) is associated with negative life outcomes and recent studies have linked it to increased mortality. These studies have examined nationwide registers or clinic-referred samples and mostly included participants up until the age of 30. No studies have investigated mortality associated with subthreshold levels of ADHD symptoms. Our aim was to analyze mortality in a perinatal risk cohort of 46-year-old adults with childhood ADHD (cADHD) and milder childhood attention problems (including hyperactivity and inattention; cAP) compared with a group with similar birth risks but no or low levels of childhood ADHD symptoms (Non-cAP). Causes of death obtained from a national register were examined.

**Methods:**

Mortality was analyzed with Cox proportional hazard models for all-cause mortality, cause-specific mortality (natural and unnatural causes), and age-specific mortality (under and over age 30). All models were adjusted with gender. The total *n* in the study was 839 (cADHD *n* = 115; cAP *n* = 216; Non-cAP *n* = 508).

**Results:**

By the age of 46, 11 (9.6%) deaths occurred in the cADHD group, 7 (3.2%) in the cAP group, and 20 (3.9%) in the Non-cAP group. The cADHD group had the highest mortality risk (adjusted hazard ratio = 2.15; 95% CI 1.02, 4.54). Mortality was not elevated in the cAP group (adjusted hazard ratio = 0.72; 95% CI .30, 1.72). Mortality in the cADHD group was mainly attributed to unnatural causes of death (adjusted hazard ratio = 2.82; 95% CI 1.12, 7.12). The mortality risk in the cADHD group was sixfold before age 30 (adjusted hazard ratio = 6.20; 95% CI 1.78, 21.57).

**Conclusions:**

Childhood ADHD was associated with a twofold risk of premature death by the age of 46 in this prospective longitudinal cohort study. Our results corroborate previous findings and the morbidity of ADHD. Subthreshold levels of childhood ADHD symptoms were not linked to increased mortality. Our results suggest that mortality risk is higher in young than middle adulthood. Future studies should examine mortality associated with ADHD in different ages in adulthood to identify those in greatest risk of premature death.

## Background

Attention-deficit/hyperactivity disorder (ADHD) is a neurodevelopmental disorder affecting 3%-5% of children [1] and approximately 2.8% of adults [2]. ADHD is associated with several impairing life outcomes, including higher rates of incarceration, suicide attempts, drug abuse, and comorbid psychiatric disorders [1, 3–5].

The association of ADHD and increased mortality has gained more research focus in recent years. Studies on this association are yet few and have mainly been large nationwide studies (Table 1). These studies have discovered an association between ADHD and increased mortality based on an ADHD diagnosis retrieved from national registers [6–8] and self-reports [9]. Others have shown ADHD to be associated with reduced life expectancy based on both register data and a longitudinally followed sample [10, 11]. Studies examining ADHD-medicated individuals have found no association with increased mortality, which might be due to a small sample or a short follow-up period [12, 13]. A recent population-based cohort study observed a lower mortality risk associated with methylphenidate use in children and adolescents with ADHD [14]. Longitudinal prospective cohort studies examining mortality of individuals with ADHD are rare. A large cohort study found externalizing symptoms including aggression and impulsivity in childhood to be associated with increased mortality risk by the age of 46 [15]. In a population-based sample study with a mean follow-up age of 27, all-cause-mortality in individuals with ADHD was not increased compared to controls [16]. Another study following clinic-referred males with ADHD to an average age of 41 showed a significant difference in death rates between the ADHD and comparison groups [4].

**Table 1:** Previous Studies on Mortality Associated with ADHD

First author (reference)	Year	Origins of Data (country)	Type of Research Design	Total N (ADHD *n*)	ADHD deceased *n*	Age of subjects/ Follow-up period	Hazard, mortality or odds ratio (95% CI)	Main outcomes
Chen [7]	2019	National database (Taiwan)	Nationwide population-based cohort study	﻿1 931 860 excluding ADHD cases (275 980)	﻿727	Mean age 9.6, range 4–44 years	AHR 1.07 (1.00–1.17)	The ADHD group had higher overall, suicide, unintentional, and homicide mortality
Sun [8]	2019	National registers (Sweden)	Nationwide cohort study	﻿2 675 615 (86,670)	﻿424	Up to 31 years of age, mean follow-up 11 years	﻿AHR 3.94 (3.51–4.43)	Mortality associated with ADHD was higher in adults than children; cumulative psychiatric comorbidities increased mortality risk
London [9]	2016	Nationally representative survey data (USA)	Prospective sample study	﻿23 352 (unknown)	unknown (2,8% of those diagnosed)	Mean age 47.6 years, followed over 4 years in 2007–2011	AOR 1.78 (1.01–3.12)	Individuals with self-reported ADHD have greater odds of dying after controlling for age
Dalsgaard [6]	2015	National registers (Denmark)	Nationwide cohort study	﻿1 922 248 (﻿32 061)	107	Up to 32 years of age	﻿AMRR 2.07 ﻿(1.70–2·50)	ADHD was associated with increased mortality, mainly due to unnatural causes
Barbaresi [16]	2013	School and medical records (USA)	Population-based birth cohort study	﻿5718 (367)	7	Mean age at follow-up 27 years, cohort born in 1976–1982	SMR ﻿1.88 (0.83–4.26)	All-cause mortality was not increased in the ADHD group but risk of death from suicide was increased
Klein [4]	2012	Research clinic (USA)	Prospective follow-up study of clinic-referred males	385 (207)	15	Mean age at follow-up 41, subjects enrolled in 1970–1978	Not available	More individuals (all males, 7%) had died by age 41 in the ADHD group compared to a comparison group

*CI* Confidence interval, *AHR* Adjusted hazard ratio, *AMRR* Adjusted mortality rate ratio, *AOR* Adjusted odds ratio, *SMR* Standardized mortality ratio

Increased mortality associated with ADHD appears to be attributed to unnatural causes. In register studies accidents, suicides, and homicides have been the main causes of death in individuals with ADHD [6–8]. Cause-specific mortality for suicide was significantly higher for those with ADHD compared to controls in a longitudinal setting [16]. ADHD is not only associated with a higher risk of suicide but also suicidal behaviors [5, 17]. Mortality in individuals with ADHD is further increased by comorbid psychiatric disorders, especially substance use disorder, oppositional defiant disorder, and conduct disorder [6, 8].

ADHD symptoms follow a continuum and might fluctuate at different times during development causing an individual to reach a diagnostic threshold at one point in time but not at another [18–20]. ADHD symptoms that remain below the diagnostic threshold have attracted increasing interest in recent years. These subclinical symptoms, or subthreshold ADHD, have been linked to similar negative life outcomes as the full disorder, such as academic deficits and psychiatric comorbidity [19, 21, 22]. To the best of our knowledge, no studies have examined mortality associated with ADHD symptoms below the diagnostic threshold.

We investigated the mortality of individuals with childhood ADHD or subthreshold symptoms associated with perinatal risks who were prospectively followed from birth up to an average age of 46 years. A group with similar perinatal risks but no or low levels of ADHD symptoms was also studied. Causes of death retrieved from a national register were also examined.

## Methods

### Study design and participants

This study is part of a larger prospective research project following a birth cohort. A total of 1196 infants with predefined perinatal risks born in a single maternity unit in Helsinki, Finland in years 1971–1974 were included in the study. The perinatal risks have been described in detail elsewhere, and included ﻿hyperbilirubinemia, birth weight < 2000 g, Apgar score < 7, respiratory distress, maternal diabetes, hypoglycemia, septicemia, or neurological symptoms [23, 24]. Participants with severe disabilities or death before age 5 (*n* = 202) were excluded [25].

**Fig. 1:**
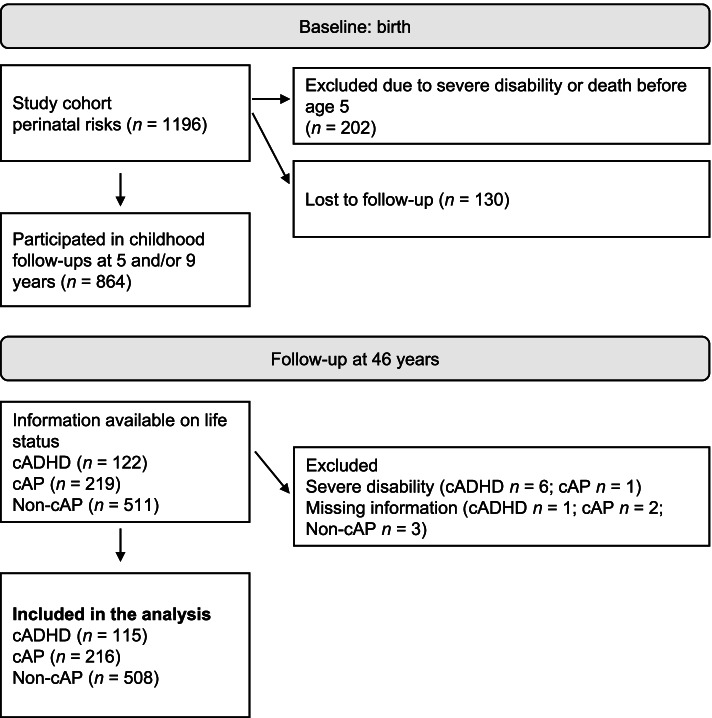
Flow chart of the participants from birth to the 46-year follow-up. Note. cADHD = childhood ADHD, cAP = childhood attention problems, Non-cAP = no childhood attention problems

The cohort was followed at 5 and 9 years of age and underwent comprehensive medical and developmental assessments [23]. Latest in-person follow-up was conducted at age 40 [26]. Participant flow during the follow-up is illustrated in Fig. 1. Comparisons between those who participated in the childhood follow-ups and those lost to follow-up are presented elsewhere [25]. Participants were excluded from the analyses if they had missing birth or childhood information or were evaluated as having a severe disability similar to the original causes for exclusion by age 5. The study group consists of 839 individuals forming three groups: childhood ADHD (cADHD, *n* = 115), childhood attention problems (cAP, *n* = 216), and no or low levels of attention problems (Non-cAP, *n* = 508). Comprehensive data from childhood follow-ups were used to form the groups, comprising questionnaire information from parents, day care, and school, and clinical assessments by a pediatrician, speech therapist, and psychologist. Questionnaires and clinical assessments gathered information on physical and psychological development and included evaluations of hyperactive and inattentive behavior. Participants with ADHD were originally classified as having minimal brain dysfunction, hyperkinetic reaction of childhood, or attention deficit [27, 28]. As ADHD did not exist in the diagnostic system during the childhood follow-ups, the principal researcher (K.M.) later retrospectively classified a total of 122 individuals as having ADHD according to Diagnostic and Statistical Manual of Mental Disorders 4th ed. criteria [29] using childhood data described above, ensuring an onset of symptoms before age 7 and persistence of over 6 months [30]. The cAP group was created based on the same childhood data, namely information from informant reports and clinical assessments. Attention problems in the cAP group include both inattentive and hyperactive symptoms and this group represents subthreshold ADHD. For an individual to be classified into the cAP group moderate ADHD symptoms had to be present in at least two settings (i.e., in day care and in one clinical assessment) or severe symptoms in one setting. The diagnostic evaluation for ADHD and forming the childhood attention problems group has been described in more detail elsewhere [26, 30]. The cADHD group had no history of ADHD medication.

### Demographical, health and mortality data

Demographical factors related to early health and environment included in the study were gender, birth weight (in grams), Apgar score at 5 min, number of perinatal risks, and childhood socioeconomic status (SES). Perinatal risks were stratified into three classes: 1, 2, and 3 or more risks (range 1–5). Childhood socioeconomic status ﻿(SES) was defined as the highest median status of mother and father recorded in childhood assessments. Four groups based on parents’ occupational level were formed with level one representing the highest status.

Information on time of death was obtained from the Population Register Centre (currently: Digital and Population Data Services Agency), Finland and on the cause of death from Statistics Finland on November 30^th^, 2019. The deceased were stratified into two age groups, under and over age 30, as previous studies have mainly consisted of individuals up to a maximum average age of 30 (Table 1). Causes of death were classified into four groups: disease, accident, suicide, and self-inflicted disease. Self-inflicted disease comprised deaths in which a harmful lifestyle was considered pivotal, e.g., death with alcoholic liver cirrhosis or acute alcoholic pancreatitis as the sole cause of death. Deaths were also stratified according to natural (disease) or unnatural (accident, suicide, and self-inflicted disease) causes of death. Information on alcohol and drug use affecting death was collected and recorded separately from the primary cause of death. Positive alcohol or drug use was recorded if the death record included a mention of substance intoxication during events prior to death or alcohol as the main reason for a disease to cause death.

### Statistical analysis

Descriptive characteristics of the study group were analyzed with chi square tests for contingency tables and ANOVAs for continuous variables. Bonferroni corrections were applied to pairwise analyses due to multiple comparisons. Effect sizes were calculated as Cramer’s *V* or partial eta squared depending on the analysis. Cox proportional hazard regression models were used to analyze survival in childhood ADHD groups. Time was calculated as years from date of birth to date of death or to the end of follow-up, November 30^th^, 2019.﻿ The unadjusted model included childhood ADHD status. Gender was entered into an adjusted model as a potential covariate. One value for SES was missing and was imputed with the median of the childhood group. Age-specific survival (under and over 30) was analyzed with separate Cox proportional hazard regression models for these age groups adjusted by gender. Cause-specific mortality for natural and unnatural causes of death in different childhood group were likewise analyzed with adjusted Cox proportional hazard regression models with gender as a covariate. Statistical analyses were conducted using IBM SPSS software, version 26.

## Results

Table 2 shows descriptive statistics for the entire study group. There were fewer males in the Non-cAP group compared to the other groups. The cADHD group had a lower mean Apgar score than the cAP group and were more likely to have more than one birth risk than the other groups. The cADHD group had more individuals in the lowest childhood SES level and less individuals in the highest childhood SES level than the other groups. Mean age of the cohort excluding the deceased was 46.9 (*SD* = 1.15; range 44–48) and there were no differences in age between the childhood groups (*p* = 0.52).

**Table 2:** Cohort characteristics

Characteristic	cADHD (1) *n* = 115	cAP (2) *n* = 216	Non-cAP (3) *n* = 508	*F / χ*^*2*^ (*df*)	*p*	*V* / *η2*	Pairwise comparison
	*M* ± *SD* or *n* (%)	*M* ± *SD* or *n* (%)	*M* ± *SD* or *n* (%)				
Gender (male)	82 (71.3%)	138 (63.9%)	238 (46.9%)	32.77 (2)	< 0.001	0.20	3 < 1,2***
Birth weight (gr)	2829.5 ± 915.7	2908.8 ± 831.8	2942.9 ± 886.7	0.80	0.45	0.002	
Apgar score	8.03 ± 2.52	8.70 ± 2.03	8.30 ± 2.31		0.02	0.01	1 < 2*
Number of birth risks				12.28 (4)	0.02	0.09	
1	52 (45.2)	133 (61.6)	308 (60.6)				1 < 2**,3*
2	43 (37.4)	65 (30.1)	147 (28.9)				
3 or more	20 (17.4)	18 (8.3)	53 (10.4)				1 > 2*
Childhood SES				35.6 (6)	< 0.001	0.15	
Level 1	11 (9.6)	45 (20.8)	113 (22.2)				1 < 2**,3*
Level 2	26 (22.6)	39 (18.1)	150 (29.5)				2 < 3**
Level 3	59 (51.3)	115 (53.2)	218 (42.9)				2 > 3*
Level 4	19 (16.5)	17 (7.9)	27 (5.3)				1 > 2*, 3***

*cADHD* Childhood ADHD, *cAP* Childhood attention problems, *Non-cAP* No childhood attention problems, *SES* Socioeconomic status

^*^ *p* < 0.05, ** *p* < 0.01, *** *p* < 0.001

A total of 38 deaths occurred in the entire cohort during the follow-up between ages 5 and 46. Nearly 10% of the cADHD group had deceased compared to 3%–4% in the other groups (Table 3). Mortality rates in different childhood groups, gender ratios, and mean age at death are presented in Table 3. There were more males in the deceased group (73.7%) than in the alive group (53.7%), χ2 (1) = 5.86, *p* = 0.016. There were no significant differences in birth weight, Apgar score, number of birth risks, or childhood SES between the deceased and alive. Seven deaths had occurred before age 30 in the cADHD group (63.6%), two in the cAP group (28.6%), and four in the Non-cAP group (20.0%).

**Table 3:** Mortality and cause of death in childhood groups

	cADHD (1)	cAP (2)	Non-cAP (3)			
Characteristic	*M* ± *SD* or *n* (%)	*M* ± *SD* or *n* (%)	*M* ± *SD* or *n* (%)	*p*	*V* / *η2*	Pairwise comparison
Deceased	11 (9.6)	7 (3.2)	20 (3.9)	0.02	0.10	1 > 2,3*
Male (deceased)	9 (81.8)	6 (85.7)	13 (65.0)	0.43	0.21	
Age (deceased)	28.86 ± 9.67	35.33 ± 8.59	37.95 ± 9.79	0.05	0.16	
Cause of death^a^						
Disease	2 (20.0)	2 (28.6)	9 (45.0)			
Accident	3 (30.0)	0 (0)	6 (30.0)			
Suicide	3 (30.0)	3 (42.9)	4 (20.0)			
Self-Inflicted disease	2 (20.0)	2 (28.6)	1 (5.0)			
Alcohol or drug related death	6 (60.0)	3 (42.9)	11 (55.0)	0.08	0.08	

*cADHD* Childhood ADHD, *cAP* Childhood attention problems, *Non-cAP* No childhood attention problems

^*^ *p* < 0.05

^a^Total *n* for cause of death = 37, 1 unknown in the cADHD group

**Fig. 2:**
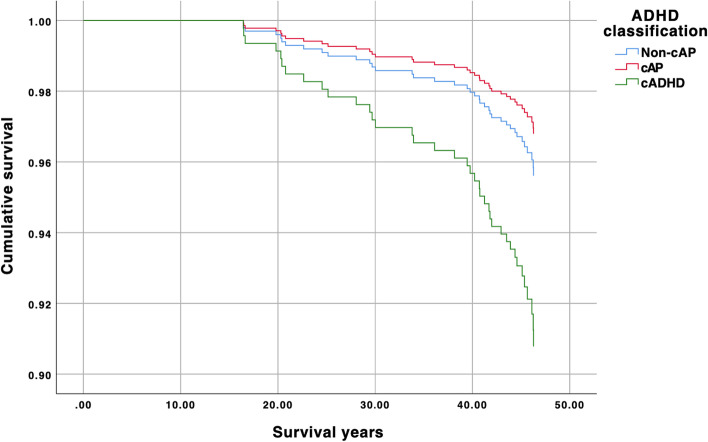
Cumulative survival in childhood groups**.** Note. cADHD = childhood ADHD, cAP = childhood attention problems, Non-cAP = no childhood attention problems

In the unadjusted Cox regression model childhood ADHD predicted survival (HR 2.53 [1.21, 5.28], *p* = 0.013). The HR remained similar for the cADHD group in the adjusted model (Table 4). Thus, the risk of death by age 46 is twofold in the cADHD group compared to the group with no childhood ADHD or attention problems. Cumulative survival in the childhood groups is illustrated in Fig. 2. Gender was a significant predictor in the adjusted model, with female gender reducing the risk of mortality (HR 0.45 [0.22, 0.95], *p* = 0.035). Due to gender being significant in the adjusted model, we added the interaction of gender and childhood group to the model, but this interaction was not significant (*p* = 0.9). Mortality risk was over sixfold in the cADHD group for age-specific mortality before age 30 (Table 4). A similar model for over age 30 did not yield significant results (HR for cADHD = 2.07 [0.79, 9.25], *p* = 0.11).

**Table 4:** Cox proportional models predicting mortality risk^a^

	Variable	HR [95% CI]	*p*
All-cause mortality	cADHD	2.15 [1.02, 4.54]	0.04
	cAP	0.73 [0.31, 1.72]	0.47
	Gender (male)	2.21 [.1.06, 4.60]	0.04
Mortality before age 30	cADHD	6.20 [1.78, 21.57]	0.004
	cAP	0.98 [0.98, 0.18]	0.98
	Gender (male)	3.51 [0.76, 16.21]	0.11
Mortality for unnatural causes of death	cADHD	2.82 [1.12, 7.12]	0.03
	cAP	0.94 [0.32, 2.72]	0.91
	Gender (male)	2.26 [0.88, 5.78]	0.09

^a^All models are adjusted with gender

*cADHD* Childhood ADHD, *cAP* Childhood attention problems, *Non-cAP* No childhood attention problems, *HR* Hazard ratio, *CI* Confidence interval

Cause of death was recorded in 37 out of 38 cases. One cause of death remained unknown in the cADHD group (death abroad resulting in register data not being available). The cause of death was unnatural in 8 out of 10 cases (80%) in the cADHD group, 5 out of 7 cases (71.4%) in the cAP group, and 11 out of 20 cases (55%) in the Non-cAP group. Causes of death in the childhood groups are presented in Table 3. The hazard ratio for mortality was nearly threefold for unnatural causes of death in the cADHD group compared to the Non-cAP group (Table 4). The cause-specific HR of 1.11 (0.23, 5.32) for natural death in the cADHD group was not significant (*p* = 0.90).

## Discussion

Childhood ADHD was associated with increased mortality in this prospective cohort study over 46 years. Deaths in the cADHD group were mostly attributed to unnatural causes and were likely to occur in young adulthood. Childhood subthreshold ADHD symptoms were not associated with increased mortality.

Childhood ADHD was associated with over a twofold increase in mortality risk compared to a group with no childhood ADHD or subthreshold symptoms up to age 46. Our results support findings from large nationwide studies that have found an association between ADHD and increased mortality [6–8]. The twofold increase in mortality is of similar magnitude as in a Danish epidemiological study, where more than double the risk of death for individuals with ADHD was discovered [6]. In contrast, a prospective sample study up to age 27 did not find increased risk of mortality in individuals with ADHD [16]. Compared to another prospective longitudinal study up to age 41, the proportion of deceased (7.2%) equaled over a twofold proportion compared to the comparison group (2.8%), and was similar to the proportion observed in this study (9.6%) [4]. It should be noted that individuals in our study were originally recruited to investigate the effects of perinatal risks on development, and ADHD symptoms were discovered during general assessments. In contrast, the other longitudinal prospective study described above [4], consists of individuals referred to a medical clinic due to ADHD symptoms, and the prospective sample study used school and medical records to attain ADHD diagnosis [16]. Our results extend the growing evidence of increased mortality associated with ADHD and broaden previous results by showing that increased mortality is also present in a longitudinally followed, non-clinic-referred, and medication-naive cohort with a follow-up of 46 years.

Those in the cADHD group were more likely to die younger than the remaining cohort with over 60% of deaths occurring before age 30. Mortality risk for under age 30 was over sixfold in individuals with childhood ADHD whereas mortality was not increased in the age group of 30 to 46 years. No deaths occurred in the cADHD group in childhood, implying that adolescence and young adulthood might be a period of high risk for those with childhood ADHD. In a Swedish register study of a population of more than 2.6 million, 40 individuals with ADHD had died before the age of 17 [8]. We did not find such an effect in our smaller cohort. Subjects with severe disabilities were excluded from our cohort, which is one possible explanation for no deaths in childhood in our follow-up. Two nationwide studies have examined mortality in different age groups and found the association between increased mortality and ADHD to be higher in adulthood than in childhood [6, 8]. These studies included adults aged 18 to 32 and thus lacked information on mortality rates later in life. This is the first study to imply that mortality risk associated with childhood ADHD might decrease after young adulthood. Further research is needed to study the effect of age on mortality in ADHD in different age groups in adulthood to identify those at greatest risk of premature death.

The cause-specific mortality risk for unnatural death was higher in the cADHD group compared to the other groups. This risk was over twofold and of similar magnitude as the all-cause mortality risk. Our results support the results of studies that have found greater risk of death due to unnatural causes, especially unintentional injuries and suicides in individuals with ADHD compared to comparison groups [4, 6–8, 16]. Interestingly, the proportion of suicides and accidents as causes of death appeared not to differ between the childhood groups, although we could not statistically analyze this due to a small sample size. Also, because of the small sample size, minor changes in the distribution of causes of death in the childhood groups could result in major changes in the relative proportions. Unnatural causes of death in this study also included diseases in which the main driver was harmful lifestyle. Thus, even though injury-related deaths (accidents and suicides) appeared not to be overrepresented in the cADHD group, nearly all deaths in this group could be attributed to behavior increasing the risk of morbidity. Consistent with our finding, ADHD has been associated with reduced estimated life expectancy and reduced healthy life expectancy partly due to common factors associated with increased mortality, such as alcohol and tobacco use [11].

We found no association between subthreshold ADHD symptoms and increased risk of mortality. Prior studies have demonstrated that subthreshold ADHD is linked to high morbidity and functional impairment similar to that of the full disorder, including psychiatric comorbidity and educational and interpersonal dysfunction [19, 21, 22]. Thus, although subthreshold ADHD symptoms pose a risk for adverse functional outcomes, risk of premature death appears not to be elevated. As this is the first study to examine mortality associated with subthreshold levels of ADHD symptoms, further research is needed before more certain conclusions can be drawn.

The cohort in this study consists of individuals with various perinatal risks, which on their own might contribute to mortality risk. Preterm birth has been linked to increased risk of mortality in early to mid-adulthood [31, 32]. A population-based cohort study found low birth weight to increase the risk of mortality in males after age 15 years [33]. Less is known about the association of other perinatal risk factors and adult mortality, but individuals with perinatal complications have been found to exhibit signs of accelerated aging at age 38 years [34]. We did not find birth weight, Apgar score, or the total number of birth risks to differ between the deceased and the remaining cohort. The cADHD group had a lower Apgar score and more birth risks than the other groups, implying that events during birth are likely to contribute to the development of the disorder itself [35].

The main strength of this study is the long follow-up that allowed the examination of mortality associated with ADHD in a prospective longitudinal setting to a later age than any previous studies. Another strength is the investigation of subthreshold ADHD symptoms. To the best of our knowledge, there are no other studies on the mortality associated with non-diagnostic levels of ADHD symptoms. The childhood ADHD group was homogenous due to similarity in perinatal and environmental background (similar perinatal risks, all born in the same hospital and living in urban environments) and due to not being medicated for ADHD. As the ADHD group was not clinic-referred for ADHD, there was no bias related to access to diagnosis.

There are some possible limitations regarding our study. Due to small sample size, we were unable to analyze the effect of all different causes of death in the childhood groups. Information on psychiatric comorbidities was not available and we could not examine comorbidities as possible confounders in mortality. However, in another study of the cohort, we did not find differences between the childhood groups in self-reported alcohol consumption or symptoms of depression and anxiety [26]. Moreover, alcohol and drug use related to death was similar in the childhood groups, implying that substance misuse was similar in the deceased. Our study cohort consists of individuals with perinatal risks from an era when ADHD medication was not available and generalizations to other populations should be made with caution.

## Conclusions

In conclusion, childhood ADHD is associated with a twofold mortality risk by age 46 in a longitudinally followed cohort of individuals with perinatal risks. This risk was higher in young adulthood and deaths were mostly attributed to unnatural causes. Subthreshold levels of childhood ADHD were not associated with increased risk of death. Our results corroborate the morbidity of childhood ADHD during the lifespan. Even though further research is needed, our results suggest that especially young adults with childhood ADHD are at greater risk of premature death, which calls for preventative measures to be aimed at this age group.

## Data Availability

The datasets generated and analyzed during the current study are not publicly available due to data sharing restrictions stated by the register owners from whom data was obtained and due to a possibility of individual privacy being compromised but are available from the corresponding author on reasonable request.

## Abbreviations

ADHD: Attention deficit hyperactivity disorder
cADHD: Childhood attention deficit hyperactivity disorder
cAP: Childhood attention problems
Non-cAP: No childhood attention problems
SES: Socioeconomic status
